# Systemic Inflammation and COVID-19 Mortality in Patients with Major Noncommunicable Diseases: Chronic Coronary Syndromes, Diabetes and Obesity

**DOI:** 10.3390/jcm10081545

**Published:** 2021-04-07

**Authors:** Andreea-Luciana Buicu, Simona Cernea, Imre Benedek, Corneliu-Florin Buicu, Theodora Benedek

**Affiliations:** 1 Doctoral School, George Emil Palade University of Medicine, Pharmacy, Science, and Technology of Târgu Mureş, 540139 Târgu Mureș, Romania; andreea_buicu@yahoo.com; 2 Department M3/Internal Medicine I, George Emil Palade University of Medicine, Pharmacy, Science, and Technology of Târgu Mureş, 38 Gheorghe Marinescu street, 540139 Târgu Mureș, Romania; 3 Diabetes, Nutrition and Metabolic Diseases Outpatient Unit, Emergency County Clinical Hospital, 540136 Târgu Mureș, Romania; 4 Clinical Department of Internal Medicine, George Emil Palade University of Medicine, Pharmacy, Science, and Technology of Târgu Mureş, 540139 Târgu Mureș, Romania; imre.benedek@umfst.ro (I.B.); theodora.benedek@gmail.com (T.B.); 5 Clinic of Cardiology, Emergency County Clinical Hospital, 540136 Târgu Mureș, Romania; 6 Public Health and Management Department, George Emil Palade University of Medicine, Pharmacy, Science, and Technology of Târgu Mureş, 540139 Târgu Mureș, Romania; florin_buicu@yahoo.com

**Keywords:** inflammation, cytokine storm, chronic coronary syndrome, type 2 diabetes, obesity

## Abstract

COVID-19 is currently considered an inflammatory disease affecting the entire organism. In severe forms, an augmented inflammatory response leads to the fulminant “cytokine storm”, which may result in severe multisystemic end-organ damage. Apart from the acute inflammatory response, it seems that chronic inflammation also plays a major role in the clinical evolution of COVID-19 patients. Pre-existing inflammatory conditions, such as those associated with chronic coronary diseases, type 2 diabetes mellitus or obesity, may be associated with worse clinical outcomes in the context of COVID-19 disease. These comorbidities are reported as powerful predictors of poor outcomes and death following COVID-19 disease. Moreover, in the context of chronic coronary syndrome, the cytokine storm triggered by SARS-CoV-2 infection may favor vulnerabilization and rupture of a silent atheromatous plaque, with consequent acute coronary syndrome, leading to a sudden deterioration of the clinical condition of the patient. This review aims to present the current status of knowledge regarding the link between COVID-19 mortality, systemic inflammation and several major diseases associated with poor outcomes, such as cardiovascular diseases, diabetes and obesity.

## 1. Introduction

The novel coronavirus disease produced by SARS-CoV-2, a virus producing severe acute respiratory syndrome, caused a global pandemic with serious medical, psychological and socioeconomic consequences [1,2,3,4]. According to official reports of the World Health Organization, a number of almost 100 million infected cases have been reported by 25 January 2021, leading to over 2 million deaths so far. The number of new infections continues to increase day by day, with the highest prevalence in America and Europe.

The most common clinical manifestations of the novel COVID-19 disease are represented by respiratory symptoms that reflect COVID pneumonia progressing, in the most severe cases, to acute respiratory distress syndrome. However, it seems that pre-existing diseases associated with chronic inflammation, such as diabetes, obesity or cardiovascular diseases (CVD), are associated with significantly worse outcomes and higher mortality after COVID-19 infection, these comorbidities being reported as the most powerful predictors of worse outcomes and death following COVID-19 disease [5].

This review aims to present the current status of knowledge regarding the link between COVID-19 mortality, systemic inflammation and several major diseases associated with poor outcomes, such as cardiovascular diseases, diabetes and obesity.

## 2. COVID-19, an Inflammatory Disease

It is currently considered that COVID-19 is mainly an inflammatory disease affecting the entire organism. The rapid elevation of proinflammatory cytokines triggered by monocyte infiltration stimulates the inflammatory response, resulting in the apoptosis of T cells, aiding in the propagation of the disease [6,7,8,9,10,11,12]. Interestingly in this disease, the inflammatory response may still increase, although the viral load is on a lowering trend [13,14,15].

It has been demonstrated already in the first phases of the COVID-19 pandemic that the augmented inflammatory response, leading to the fulminant “cytokine storm”, may result in severe multisystemic end-organ damage. The amplitude of the cytokine storm is considered the main factor associated with systemic organ failure and death [16].

According to very recent studies, it seems that an uncoordinated immune response triggers the excessive release of cytokines and chemokines from respiratory cells and macrophages, recruiting inflammatory cells, causing excessive infiltration of the lungs and systemic organs and further amplifying immune-related end-organ damage [16].

## 3. COVID-19 Mortality and Chronic Inflammatory Status

Chronic inflammation, along with sustained exposure to glucolipotoxicity, and oxidative stress are associated with dysfunctions of both innate and adaptive immunity [17]. These include M1 macrophage polarization, a proinflammatory cytokine profile in dendritic cells (DCs), abnormal Natural Killer (NK) cell phenotypes and function, decreased naïve CD4+ T cells, a shift of helper T cells toward a proinflammatory Th17 and Th22 profile, impaired γδT cell proliferation unresponsive to epithelial damage, decreased Treg response and impaired B cell function with impaired antibody response [17]. These might in part explain the worse clinical outcome seen in COVID-19 patients with obesity, type 2 diabetes (T2DM) and CVD.

In fact, patients with severe respiratory failure with COVID-19 seem to exhibit a unique pattern of immune dysfunction: macrophage activation syndrome (MAS); interleukin (IL)-6-mediated very low human leukocyte antigen D related (HLA-DR) expression, with severe depletion of CD4+ T cells, CD19+ T cells and NK cells; and sustained production of cytokines and hyperinflammation [18]. Part of the cytokine storm induced by the coronavirus infection is the master IL1-β, which has been shown to be produced via NOD-like receptor (NLR) family pyrin domain containing (NLRP)3 inflammasome/NF-kB activation [19]. This, in turn, stimulates the secretion of other proinflammatory cytokines, thus amplifying the cytokine storm [20]. The hyperproduction of proinflammatory cytokines, such as IL-1, IL-6, IL-12, interferon γ (IFN-γ) and tumor necrosis factor α (TNF-α), which preferentially target the pulmonary tissue, seems to significantly worsen the prognosis of COVID-19 patients [20,21]. In addition, patients with high IL-1β levels presented hypercoagulation and disseminated intravascular coagulation [22].

In fact, COVID-19 presents a unique and inappropriate inflammatory response, defined by decreased levels of type I and III interferons and increased production of IL-6 and chemokines (that attract/recruit T cells, NK cells, monocytes and/or macrophages and neutrophils) [23]. Patients with pre-existing comorbidities might present this undesirable inflammatory response more frequently, and this might explain the worse clinical outcomes in such cases [20].

Chronic inflammation and immune dysregulation are only a part of the picture. Another piece of the puzzle is endothelial dysfunction, caused in part by chronic inflammation [24]. Reports indicate the presence of viral elements of SARS-CoV-2 in endothelial cells and the accumulation of inflammatory cells, causing endothelial cell injury and death in several organs, which explains the systemic impaired microcirculatory function [25]. Patients with pre-existing endothelial dysfunction, such as those with T2DM, obesity or CVD, might therefore have worse clinical outcomes in the context of COVID-19 infection [26]. In addition, cardiac abnormalities, a prothrombotic environment and increased blood viscosity are other possible mechanistic explanations for these unfavorable outcomes [26,27].

Indeed, two meta-analyses of 30 studies (6452 patients) and 33 studies (16,003 patients), respectively, showed that diabetes was associated with a higher risk of mortality (RR (risk ratio): 2.12 (95% confidence interval (CI): 1.44, 3.11), *p* < 0.001; pooled OR (odds ratio): 1.90 (95%CI: 1.37–2.64), *p* < 0.01) with COVID-19 infection [28,29]. Another subsequent meta-analysis (76 studies; 31,067 subjects) was basically in line with previous reports: COVID-19 patients with diabetes had higher mortality rates (28.5 vs. 13.3%, respectively; *p* < 0.01) and higher mortality risk (OR: 2.21 (95%CI: 1.83–2.66), *p* < 0.001) [30].

Similarly, an analysis of a large database from Mexico (177,133 subjects) indicated that patients with diabetes had a particularly higher mortality rate (21.8% vs. 7.7%) and that higher mortality risk was found for early-onset diabetes (<40 years of age) (HR (hazard ratio): 2.754 (95%CI: 2.259–3.359)) [30]. The same analysis showed that obesity was the only condition, which conferred an increased mortality risk exclusively for COVID-19 vs. non-COVID-19 (HR: 1.261 (95%CI: 1.109–1.433)) [31].

Obesity turned out to be another condition that is associated with a higher risk of fatal outcomes in COVID-19 patients. A systematic review and meta-analysis of 30 studies with body mass index (BMI)-defined obesity and 3 controlled studies with visceral adipose tissue (VAT)-defined adiposity (45,650 subjects) demonstrated by multivariate analyses higher ORs of COVID-19-associated mortality with higher BMI (1.49 (95%CI: 1.20, 1.85), *p* < 0.001) [32]. Other meta-analyses basically reported similar mortality risk ratios in patients with obesity (OR: 1.65 (95%CI: 1.21–2.25); *p*: 0.001; OR: 1.57 (95%CI: 0.85–2.90)), but morbid obesity was linked with a greater COVID-19 mortality risk (OR: 3.76 (95%CI: 2.67–5.28)) [33,34].

Data in the literature also indicate that COVID-19 patients with underlying CVD and hypertension might face a higher risk of mortality. The in-hospital mortality in patients with COVID-19 was greater in the presence of CVD and hypertension (unadjusted OR: 4.85 (95%CI: 3.07–7.70); and 3.67 (95%CI: 2.31–5.83), respectively) [35]. CVD was associated with a significantly higher risk of mortality from COVID-19 infection in another meta-analysis that investigated the association of pre-existing comorbidities with COVID-19 mortality (RR: 2.25 (95%CI: 1.60–3.17)) [36].

## 4. Cardiovascular Dimension of COVID-19

The cardiovascular system seems to present extremely complex interactions with COVID-19 disease. Coronary artery disease, heart failure, hypertension and various forms of cardiac arrhythmia are the most common types of CVD reported in patients with severe forms of SARS-CoV-2. However, the clinical presentation of CVD in patients infected by SARS-CoV-2 may be very heterogenous. Importantly, cardiac involvement is possible even in the absence of any respiratory symptoms [37].

Myocardial injury is an important pathogenic feature of COVID-19 disease [1,2,13,38,39,40]. Myocarditis, heart failure, acute myocardial infarction, arrhythmia, cardiac arrest or myocardial infarction with nonobstructive coronary arteries (MINOCA) were observed in a significant proportion of patients with SARS-CoV-2. At the same time, mortality associated with myocardial infarction is higher in the context of coexisting SARS-CoV-2 infection.

Hospitalized patients with COVID-19 disease and concomitant cardiac disease have an extremely poor prognosis compared to those without a cardiac disease [41]. Therefore, cardiovascular patients seem to require careful monitorization in the case of SARS-CoV-2 infections. At the same time, all cardiovascular manifestations that appear in COVID-19 patients, regardless of their comorbidity, require further investigations, close monitoring, adequate and prompt treatment in order to prevent the development of severe cases and death.

## 5. CVD and COVID-19 in Children

The recent occurrence of new variants of SARS-CoV-2 has led to a strong interest in regard to the pediatric diffusion of the virus. The pediatric population may present with different clinical patterns associated with COVID-19 infection [42]. Pediatric patients with COVID-19 disease seem to have better outcomes. However, they may develop a particular form of multisystem inflammatory syndrome that includes cardiovascular manifestations, such as myocarditis, rhythm disturbance, valvular regurgitation, depressed myocardial function and coronary artery inflammation [43,44]. Genetically, underexpression of the ACE2 receptor in children infected with SARS-CoV-2 may further downregulate the ACE2 expression, leading to an increase in inflammation and progression to Kawasaki-like disease [45].

In children, even mild cases of infections with SARS-CoV-2 can present multisystem inflammatory syndrome with Kawasaki-like symptoms, with severe evolution, even in the absence of any metabolic syndrome [46]. A distinct type of inflammation described as Kawasaki-like multisystem inflammatory syndrome temporally associated with SARS-CoV-2 may lead to a life-threatening condition in previously healthy children and adolescents [43,47,48]. Coronary artery aneurysms appear to be an important complication in children admitted to an intensive care unit with COVID-19. At the same time, myocardial injury outside Kawasaki syndromes has been reported in pediatric patients infected with SARS-CoV-2, and a recent case report describes reversible myocardial injury associated with COVID-19 in an infant. All these show that COVID-19 screening should be performed in children with myocardial injury in an inflammatory context [49,50].

Although immediate survival seems to be high among children with SARS-CoV-2 infections, long-term outcomes of this condition are still unknown [14,51,52].

## 6. Inflammation, Acute Coronary Syndromes and COVID-19

The link between chronic inflammation and CVD is already well known, as well as the role of inflammation in triggering plaque vulnerabilization and consequently, an acute coronary event [53,54]. In COVID-19, this link seems to be further responsible for the worse outcomes reported in cardiovascular patients. At the same time, increased inflammation triggered by SARS-CoV-2 infection may be also responsible for the higher rate of acute coronary syndromes encountered in the post-COVID period because many patients healed from COVID-19 return to the emergency room after several weeks, for acute chest pain caused by the rupture of an atherosclerotic plaque [55].

A persistent increase in systemic inflammation, such as in periodontal disease or following gut microbiota alteration, has been associated with a higher risk of atheromatous plaque initiation and progression [56,57]. At the same time, systemic inflammation, as well as the local inflammation at the site of coronary arteries, may favor plaque rupture and coronary thrombosis. Interestingly, it has been suggested that the epicardial fat (EF) surrounding coronary arteries, which is currently known to be associated with cardiovascular events via EF-mediated inflammation, may play a significant role in COVID-19 disease, acting as a reservoir of the SARS-CoV-2 virus. An increase in the volume of epicardial fat has been associated with worse outcomes of this disease, suggesting that epicardial fat may contribute to COVID-19 myocardial inflammation [58].

Acute coronary syndrome (ACS) is caused, in most cases, by thrombotic complications occurring at the site of an atherosclerotic plaque. Systemic inflammation has been demonstrated to play a significant role in plaque destabilization in patients with ACS, and inflammatory biomarkers are usually higher in patients with ACS than in those with stable coronary disease. Inflammatory biomarkers such as C-reactive protein (CRP) presented significantly higher values in the days following an acute coronary event. At the same time, the amplitude of systemic inflammation expressed by the serum levels of hs-CRP was strongly correlated with long-term outcomes after acute coronary syndrome [57]. Therefore, it is clear that systemic inflammation caused by a viral infection could play a key role in the pathogenesis of acute coronary syndromes [55]. This is also valid for SARS-CoV-2 infections due to similar mechanisms and incidence of acute coronary syndromes among COVID-19 patients [5,7].

**Figure 1:**
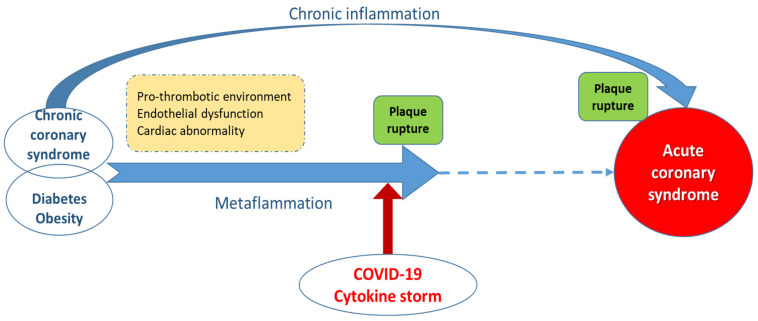
The link between COVID-19 cytokine storm and acute coronary syndrome (ACS). Usually, ACS is produced by the rupture of an atheromatous plaque, after a long evolution of a chronic coronary syndrome, which may be associated with persistent, long-term inflammation. The sudden augmentation of systemic inflammation in the context of cytokine storm initiated by COVID-19 disease may trigger early rupture of a pre-existing plaque and consequent ACS.

Figure 1 illustrates the link between COVID-19 cytokine storm and ACS. Usually, ACS is produced by the rupture of an atheromatous plaque after the long evolution of a chronic coronary syndrome, which may be associated with persistent, long-term low-grade inflammation. The sudden augmentation of systemic inflammation in the context of cytokine storm initiated by COVID-19 disease may trigger the early rupture of a pre-existing plaque and consequent ACS.

The increase in inflammatory cells due to respiratory viruses leads to the increased production of cytokines, proteases and vasoactive molecules that produce endothelial damage. This endothelial damage exposes the content of the subendothelial space to circulating prothrombotic factors and initiates thrombus formation. The destabilization of stable atheromatous plaques and the complex process of plaque vulnerabilization, as well as rapid progression of new plaques, are involved in the cardiovascular complications in COVID-19 patients, leading to direct myocardial damage and cytokine storm [59,60,61,62].

At the same time, inflammation at the myocardial level can cause myocarditis, heart failure, cardiac arrhythmias, acute coronary syndrome, rapid deterioration and sudden death [5]. It was clearly demonstrated that previous coronary artery disease may modulate cardiac damage in COVID-19 patients. A study by Schiavone et al. found myocardial injury, defined by elevated serum levels of high-sensitivity cardiac troponin (hsTn), in 43.8% of COVID-19 patients with coronary artery disease compared to only 14.4% in COVID-19 patients without coronary artery disease [63]. This was also reflected in a higher mortality rate in the group with coronary artery disease, similar to other studies that identified a higher mortality rate in patients with elevated cardiac troponins and established the prognostic significance of cardiac injury in COVID-19 patients [63].

## 7. Biomarkers Associated with Worse Prognosis in COVID-19 Patients Suffering Acute Coronary Syndromes

The most frequently used blood tests useful in COVID-19 patients with cardiovascular involvement are: N-terminal (NT)-pro Brain Natriuretic Peptide (BNP)/BNP, troponin, D-dimer, procalcitonin, full blood count, IL-6 and ferritin. Biomarkers indicating rapid deterioration in COVID-19 patients are mostly represented by troponin I (TnI), CRP, erythrocyte sedimentation rate (ESR), D-dimer, progressive deterioration of lymphocyte counts and elevated inflammatory markers (IL-6, TNF-α). The cytokine storm is characterized by elevated levels of cytokines, mainly interleukins IL-6, IL-7 and IL-22. Relevant findings from COVID-19 studies related to inflammatory markers are presented in Table 1.

Acute myocardial injury, caused by myocardial infarction or myocardial inflammation, is associated with increased serum levels of hsTn. A very recent study indicated that a rise in hsTnI was an independent predictor for mortality in COVID-19 patients, with a four-fold increase in the risk of death (OR: 3.84 (95%CI: 1.78–8.28)) [74].

Not all troponin elevations are caused by acute coronary syndromes. The elevation of simple cardiac biomarkers may be misleading, especially in the setting of chronic coronary syndrome, because this biomarker is mostly influenced by baseline characteristics, such as pre-existing coronary artery disease [63,75,76]. Troponin is also increased in other clinical conditions, such as heart failure, respiratory insufficiency or renal failure. Constant elevations of serum troponin do not necessarily reflect irreversible myocardial injury resulting from ischemic or inflammatory injury [77]. However, in COVID-19 patients, troponin levels progressively increased in parallel with the development of multisystemic organ failure, and a sudden increase in hsTN levels after 10–14 days of evolution seems to be associated with reduced survival [78].

Pro-BNP is another biomarker used along with Tn to identify and evaluate acute cardiac damage. Patients with acute or chronic coronary syndromes presenting with CODIV-19 without myocardial involvement may show Tn elevation but normal levels of pro-BNP, while cases with myocardial involvement show elevated pro-BNP along with Tn. Thus, proBNP may help to discriminate COVID forms associated with myocardial involvement from those without myocardial damage. Natriuretic peptides may also reflect the severity of the COVID-19 disease. One study showed that both NT-proBNP and hs-cTnI were significantly higher in critical COVID-19 cases [79]. Another study found that higher levels of Tn and NT-proBNP were associated with the severity and case fatality rate of COVID-19 [80].

At the same time, coagulation abnormalities are among the major determinants of CV involvement in COVID-19, and coagulation biomarkers are associated with increased morbidity and mortality in COVID patients. The pathway from COVID-19 infection to macro or microthrombosis may be linked to the cytokine storm, especially in patients with chronic coronary syndromes. This effect is related not only to the higher risk of coronary thrombosis but also to the increased rate of venous thromboembolism, which is quite frequent in SARS-CoV-2 infection. Hemostatic abnormalities associated with this condition lead to pulmonary microthrombosis, intravascular coagulopathy and myocardial injury, being reflected by elevated levels of cardiac biomarkers, D-dimers, fibrin degradation products and prolonged prothrombin time [81,82]. D-dimers may predict vascular involvement, coagulopathy and or silent microthrombosis, D-dimer elevation being associated with worse outcomes. However, the role of D-dimers in the clinical management of patients with severe forms of COVID-19 should be further analyzed, as well as the initiation of subsequent anticoagulant therapy in these severe cases.

A strategy of the early evaluation and continuous monitoring of cardiac damage (based on troponin and NT-proBNP values) and coagulation status (based on D-dimer levels) may identify patients with cardiac injury and predict COVID-19 complications [2]. High levels of IL-6, IL-1B and IL-8 have been associated with plaque instability and increased thrombotic risk, contributing to the initiation of ACS. In severe cases of COVID-19, high levels of TNF-α, IL-1 and IL-6 are associated with coagulation activation and thrombin generation, leading to disseminated intravascular coagulation or thrombotic complications.

The most relevant biomarkers used to monitor clinical progression and their association with different types of cardiovascular complications in COVID-19 patients are listed in Table 2.

## 8. Statins and COVID-19 Disease Progression

Statins act at different levels in the inflammation-mediated atherosclerotic process. For instance, statins regulate inflammasome-mediated inflammation, being involved in the critical pathway of inflammation-mediated effects of COVID-19. It is well known that statins inhibit HMG-CoA reductase, which leads to inhibition of cholesterol biosynthesis. At the same time, COVID-19 infection induces lipid raft alterations of the host cells involved in viral replication. Therefore, it has been suggested that statins acting at the level of lipid rafts and disrupting them could reduce viral entry into the cells, viral replication and cell infection [83].

Other studies indicate that statins upregulate the expression of ACE2, which is associated with increased viral entry into the cells. Moreover, low-serum low-density lipoprotein (LDL) cholesterol levels seem to be associated with a higher risk of infection [84]. A recent study on 154 subjects with COVID-19 subjects failed to show a significant favorable effect of statins [85]. Other studies clearly indicated that statin therapy did not show favorable effects on COVID-19 outcomes, suggesting that prior statin therapy may be considered as a proxy of underlying comorbidities, which increase the risks of more severe clinical presentations [86].

All these indicate that the effect of statins on COVID-19 patients is more complex, has not been fully elucidated so far, and is still controversial.

## 9. Chronic Inflammation in Obesity and Type 2 Diabetes

T2DM is associated with chronic, low-grade inflammation, referred to as metabolism-induced inflammation (“metaflammation”), along with a plethora of metabolic, hormonal and molecular alterations, leading to the initiation and/or progression of the disease itself and to its associated chronic complications [87,88,89]. In fact, diabetes, obesity and CVD are part of a cluster of diseases associated with metaflammation and immunometabolic dysfunction, leading to disability, premature aging and death [88]. Apart from the adipose tissue and pancreas, many other organs and cells are involved in this immunometabolic interconnection, i.e., liver, brain, gut, skeletal muscles, innate and adaptive immune systems [87,88].

It has been long recognized that adipose tissue is a source of inflammatory cytokines and chemokines (e.g., TNFα, IL-6, monocyte-chemoattractant protein 1 (MCP-1)) that are associated with insulin resistance by interfering with key steps of the insulin signaling pathway [90,91,92]. More recent data showed that not only are adipocytes of the white adipose tissue (WAT) a source of proinflammatory cytokines but also the infiltrated macrophages, which suffer M1/M2 (proinflammatory/anti-inflammatory) polarization in obesity. They play a role not only in insulin resistance/T2DM but also in CVD [93]. It is apparent that M1/M2 macrophages show differences in their cellular metabolisms, as the M1 macrophages present increased aerobic glycolysis and inflammation (that block insulin action), while the M2 macrophages rely on β-oxidation of fatty acids to extract energy [89,94,95,96,97]. Of course, there are various intermediate phenotypes, as macrophages are a heterogeneous cell population [94]. The same distinctive metabolic profiles are exhibited by other cell types involved in the inflammatory response, i.e., Th1, Th17 cells, activated microglia or endothelial cells, which produce highly demanded energy through glycolysis upon inflammation, while regulatory T cells (Tregs), memory T cells and resting microglia rely on fatty acid oxidation to maintain tissue homeostasis [98,99]. The effector T cells (as well as M1 macrophages) express high levels of glucose transporter 1 (GLUT1) and are highly glycolytic, while the Treg express low levels of GLUT1 [99]. Increased glycolysis fuels inflammation through various molecular mechanisms, such as signaling modulation, post-transcriptional and post-translational modification, epigenetic regulation, that trigger the expression of proinflammatory gene expression and cytokines secretion [99].

Thus, stimuli such as IFN-γ, IL-1β or lipopolysaccharide (LPS), along with changes in the intracellular metabolism of immune cells, may determine alterations in the function and stimulation of the proinflammatory response [88,94].

There is a large body of evidence showing that changes in nutrient availability have an important impact on the immune response. In vitro and animal studies demonstrated that hyperglycemia is associated with the increased production of proinflammatory cytokines in the immune cells [100,101,102]. Postprandial hyperglycemic spikes are associated with a transient proinflammatory response via macrophage-derived IL-1β, which also stimulates insulin secretion and glucose disposal [103]. Moreover, hyperglycemia seems to be involved in the “trained immunity” (the nonspecific, immunological memory of past insults) through epigenetic modifications that sustain inflammation and transcriptional activation of genes involved in atherosclerosis [101,104]. Interestingly, on the other hand, heparin inhibits the proinflammatory polarization of macrophages in hyperglycemic conditions and promotes an anti-inflammatory phenotype of these cells [105].

The molecular mechanisms through which hyperglycemia induces inflammation are complex. Part are due to reactive oxygen species (ROS) production and oxidative stress, activation of inflammatory kinases and toll-like receptors (TLR), NF-kB signaling, nonenzymatic glycation, etc. [88,106]. In addition, various epigenetic modifications, such as alterations of the microRNA expression, histone methylation/acetylation, may occur, which change the expression of proinflammatory genes [107]. Hyperglycemia may also favor the accumulation of senescent cells in various tissues that exhibit a proinflammatory profile [88,108].

Nutrients also play a role in immunometabolism. Saturated fatty acids (SFAs) (mainly palmitic, lauric and stearic acids) stimulate inflammatory pathways through the activation of TLRs, NF-kB, ROS production, etc., while omega-3 fatty acids (docosahexaenoic acid) have opposite effects by inhibiting the TLR-mediated signaling pathways, the reduction of NF-kB activation and cytokine secretion [88,109,110,111]. The key driver of metaflammation induced by SFA is NLRP3 inflammasome (via TLR) that favors the proinflammatory IL-1β production [112,113]. Cholesterol is another important contributor to the proinflammatory response in various cell types and the metaflammation in obesity, T2DM and atherosclerosis [88,114,115]. Cholesterol crystals and the oxidized LDL cholesterol trigger the NRLP3 activation via ROS, which, in turn, increased IL-1β secretion [116,117]. The activated NLRP3-IL-1β axis seems to play an important role in the metaflammation-associated metabolic disorders, including T2DM, nonalcoholic fatty liver disease and atherosclerosis [114,115]. The oxidized LDL cholesterol may also induce trained immunity, as it causes, through epigenetic modifications, a long-lasting proatherogenic macrophages phenotype [118].

Additionally, the literature indicates that the low-grade chronic inflammation in metabolic and CVD is fueled by intestinal dysbiosis and barrier dysfunction [115,119]. Alterations in the gut microbiome (modulated by diet, intestinal immune system, biliary and pancreatic secretions) may cause metabolic derangements, the production of proinflammatory mediators by local immune cells and the dysfunction of intestinal barriers, which further favors the translocation of bacterial metabolites and pathogen-associated molecular patterns (PAMPs), such as LPS, and endotoxemia [120]. LPS drives chronic inflammation by boosting inflammasome activation [121]. Patients with T2DM have increased plasma levels of LPS, which may partially contribute to the low-grade chronic inflammation [121]. Hyperglycemia per se, on the other hand, might also induce barrier dysfunction via the epithelial GLUT-2-dependent reprogramming of the cell transcriptome [122]. Moreover, immune cells primed in the gut might be activated and recruited into distant tissues (VAT and liver), further modulating the metaflammation [115,123].

Thus, the relationship between immunity/inflammation and metabolism/atherosclerosis is complex and bidirectional, as metabolic imbalance drives chronic inflammation and the dysregulation of immune cell function, which, in turn, contribute to metabolic disturbances and the development of vascular complications.

## 10. Metabolic Dimension of COVID-19

Data published in the literature so far point to metabolic diseases, mainly diabetes mellitus and obesity, as conditions associated with more severe outcomes, including higher mortality risk, in the setting of COVID-19 [28,29,30,31,32,33,34]. These seem to be driven by both metabolic and inflammatory/immune disturbances, such as insulin resistance, prolonged, uncontrolled hyperglycemia (not just a history of diabetes mellitus), low-grade chronic inflammation, along with other factors associated with these conditions (increased glucose variability, endothelial dysfunction, hypercoagulability, etc.) [124,125].

Indeed, diabetes mellitus and/or hyperglycemia (as well as severe obesity) were most frequent in hospitalized patients with COVID-19 and, in those admitted to intensive care units, increased the hospital length of stay and likelihood of severe complications (i.e., acute respiratory distress syndrome, requiring mechanical ventilation) and mortality risk [126,127,128,129]. While hyperglycemia and high glucose variability worsen outcomes in the context of COVID-19, well-controlled blood glucose, with lower glucose variability, on the other hand, seemed to be associated with markedly lower mortality risk (adjusted HR: 0.14) [126,129,130]. Thus, the metabolic imbalances are potential contributors to a more severe clinical course of COVID-19, and perhaps they need to be addressed carefully, in a timely manner, in this context.

## 11. Conclusions

COVID-19 is associated with intense multisystemic inflammation in both adults and children, leading, in the most severe cases, to a cytokine storm that is responsible for poor outcomes. Systemic inflammation associated with COVID-19 is directly associated with cardiovascular complications. At the same time, systemic conditions associated with an increased inflammatory response, such as diabetes, obesity or chronic coronary syndromes, are responsible for worse clinical evolution and poorer outcomes in this group of patients. Data in the literature are congruent in identifying diabetes, obesity and cardiovascular disease as comorbidities associated with greater mortality risk in patients with COVID-19 infection, which might need special monitoring, tailored infection prevention, and earlier intervention to improve survival.

However, further studies are required to elucidate the complex pathophysiologic mechanisms involved in the link between chronic inflammation and COVID-19 mortality.

## Figures and Tables

**Table 1:** Chronic inflammatory markers in COVID-19 infection.

Biomarker	Reference	Study/Patients Characteristics	Study Findings
IL-6	Galván-Román, J.M. et al. [64]	146 patients; 66% males; median age: 63 years	Baseline levels >30 pg/mL predicts invasive mechanical ventilation requirement (OR: 7.1; *p* < 0.001)
	Liu, F. et al. [65]	140 patients; 35% males; age: 65.5 (54.3–73.0) years	IL-6 >32.1 pg/mL predicted severe complications (HR: 2.375; 95%CI: 1.058–5.329; *p* < 0.001)
	Gao, Y. et al. [66]	43 adult patients; age 45.20 years (severe group) and 42.96 years (mild group); 26 males	Independent risk factor for the severity (OR: 17.304 (95%CI: 2.416, 123.933); *p* = 0.005)
	Qin, C. et al. [67]	452 patients (286 with severe infection); median age: 58 years; 235 were males	Increased levels in severe vs. nonsevere cases (25.2 vs. 13.3 pg/mL; *p* < 0.001)
	Henry, B.M. et al. [68]	MA of 21 studies (*n* = 3377 patients)	Higher levels in severe vs. nonsevere disease (WMD: 1.70 (0.8, 2.6) pg/mL); significantly greater increases in nonsurvivors vs. survivors (WMD: 4.6 (3.4, 5.8] pg/mL)
	Coomes, E.A. et al. [69]	MA of 10 studies (*n* = 1798)	2.9-fold higher levels in patients with complicated disease compared with patients with noncomplicated disease; Patients requiring ICU admission had higher values (random of means: 3.24 (95%CI: 2.54, 4.14]).
	Zeng, F. et al. [70]	MA of 16 studies (*n* = 3962)	Nonsevere group had lower levels (WMD: −21.32 ng/L (95%CI: −28.34, −14.31), *p* < 0.001)
	Akbari, H. et al. [71]	MA of 44 studies (*n* = 7865)	Higher levels in severe groups (WMD: 17.79 pg/mL (95%CI: 14.24, 21.33), *p* < 0.001)
CRP	Liu, F. et al. [65]	140 patients; 35% males; age: 65.5 (54.3–73.0) years	CRP >41.8 mg/L predicted severe complications (HR: 4.394 (95%CI: 1.924–10.033); *p* < 0.001)
	Qin, C. et al. [67]	452 patients (286 with severe infection); median age: 58 years; 235 were males	Increased levels in severe vs. nonsevere cases (57.9 vs. 33.2 mg/L; *p* < 0.001)
	Mo, P. et al. [72]	155 patients (45.2% refractory patients); age: 54 (42–66) years; 55.5% males	Increased levels in refractory cases (46 (22–106) mg/L vs. 23 (10–47) mg/L; *p* = 0.001)
	Zhang, J.J. et al. [73]	140 patients; age: 57 (25–87) years; 50.7% males	Increased levels in severe vs. nonsevere cases (47.6 (20.6–87.1) mg/L vs. 28.7 (9.5–52.1) mg/L, *p* < 0.001)
	Zeng, F. et al. [70]	MA of 16 studies (*n* = 3962)	Nonsevere group had lower levels (WMD: −41.78 mg/L (95%CI: −52.43, −31.13), *p* < 0.001)
	Akbari, H. et al. [71]	MA of 44 studies (*n* = 7865)	Higher levels in severe groups (WMD: 41.07 mg/L (95%CI: 29.76, 52.38), *p* < 0.001)
Procalcitonin	Liu, F. et al. [65]	140 patients; 35% males; age: 65.5 (54.3–73.0) years	PCT >0.07 ng/mL predicts severe complications (HR: 4.908 (95%CI: 1.797, 13.402), *p* = 0.002)
	Qin, C. et al. [67]	452 patients (286 with severe infection); median age: 58 years; 235 were males	Increased levels in severe vs. nonsevere cases(0.1 vs. 0.05 ng/mL; *p* < 0.001)
	Zhang, J.J. et al. [73]	140 patients; age: 57 (25–87) years; 50.7% males	Increased in severe vs. nonsevere cases (0.1 (0.06–0.3) ng/mL vs. 0.05 (0.03–0.1) ng/mL; *p* < 0.001)
	Zeng, F. et al. [70]	MA of 16 studies (*n* = 3962)	Nonsevere group had lower levels (WMD: −0.13 ng/mL (95%CI: −0.20, −0.05), *p* < 0.001)
	Akbari, H. et al. [71]	MA of 44 studies (*n* = 7865)	Higher levels in severe groups (WMD: 0.07 ng/mL (95%CI: 0.05, 0.09), *p*< 0.001)
Ferritin	Qin, C. et al. [67]	452 patients (286 with severe infection); median age: 58 years; 235 were males	Increased levels in severe vs. nonsevere cases (800.4 vs. 523.7 ng/mL; *p* < 0.001)
	Henry, B.M. et al. [68]	MA of 21 studies (*n* = 3377 patients)	Discriminate between severe vs. nonsevere disease (WMD: 408.28 (311.12, 505.44) ng/mL); significantly greater increases in nonsurvivors vs. survivors (WMD: 760.2 (560.84, 959.53) ng/mL)
	Zeng, F. et al. [70]	MA of 16 studies (*n* = 3962)	Nonsevere group had lower levels (WMD: −398.80 mg/L (95%CI: −625.89, −171.71), *p* < 0.001)
	Akbari, H. et al. [71]	MA of 44 studies (*n* = 7865)	Higher levels in severe groups (WMD: 594.25 μg/L (95%CI: 438.10, 750.39), *p* < 0.001)
ESR	Zeng, F. et al. [70]	MA of 16 studies (*n* = 3962)	Nonsevere group had lower levels (WMD: −8 mm/h (95%CI: −14, −2), *p* = 0.005)
	Akbari, H. et al. [71]	MA of 44 studies (*n* = 7865)	Higher levels in severe groups (WMD: 23.39 mm/h, (95%CI: 16.51, 30.27), *p* < 0.001)
TNF-α	Akbari, H. et al. [71]	MA of 44 studies (*n* = 7865)	Higher levels in severe groups (WMD: 0.24 pg/mL (95%CI: 0.01, 0.47), *p* < 0.001)

IL-6 = interleukin-6; OR = odds ratio; HR = hazard ratio; CI = confidence interval; ICU = intensive care unit; MA = meta-analysis; WMD = weighted mean difference; CRP = C-reactive protein; PCT = procalcitonin; ESR = erythrocyte sedimentation rate; TNF-α = tumor necrosis factor-alpha.

**Table 2:** Systemic biomarkers associated with cardiovascular involvement in COVID-19 patients and their association with different types of complications.

Biomarkers of Cardiovascular Involvement in COVID-19 Patients	Markers of Rapid Deterioration in Adult Patients with COVID-19	Associated with the Cytokine Storm	Associated with Cardiac Damage and Coagulation and Predicts Complications	Markers of Plaque Instability and Increased Thrombotic Risk	Associated with Coagulation Activation and Thrombin Generation	Markers of Inflammation and Cardiac Involvement in Children
NT-proBNP/BNP			✓			✓
Troponin	✓		✓			✓
D-dimer	✓		✓			✓
Procalcitonin						✓
Ferritin						✓
IL-6	✓	✓		✓	✓	
C-reactive protein	✓					✓
ESR	✓					
Deterioration of lymphocyte counts	✓					
TNF-α	✓				✓	
IL-7		✓				
IL-22		✓				
CXCL10		✓				
IL-1B				✓		
IL-8				✓		
IL-1					✓	
CK						✓

✓: indicates the association between the biomarker and respective cardiovascular complication.

## References

[1] Hendren N.S., Drazner M.H., Bozkurt B., Cooper L.T. (2020). Description and Proposed Management of the Acute COVID-19 Cardiovascular Syndrome. Circulation.

[2] Guzik T.J., Mohiddin S.A., Dimarco A., Patel V., Savvatis K., Marelli-Berg F.M., Madhur M.S., Tomaszewski M., Maffia P., D’Acquisto F. (2020). COVID-19 and the cardiovascular system: Implications for risk assessment, diagnosis, and treatment options. Cardiovasc. Res..

[3] Wang D., Hu B., Hu C., Zhu F., Liu X., Zhang J., Wang B., Xiang H., Cheng Z., Xiong Y. (2020). Clinical characteristics of 138 hospitalized patients with 2019 novel coronavirus-infected pneumonia in Wuhan, China. JAMA.

[4] Coronavirus Resource Center, Johns Hopkins University. 13 April 2020. https://coronavirus.jhu.edu/.

[5] Liu P.P., Blet A., Smyth D., Li H. (2020). The Science Underlying COVID-19: Implications for the Cardiovascular System. Circulation.

[6] Basso C., Leone O., Rizzo S., De Gaspari M., van der Wal A.C., Aubry M.C., Bois M.C., Lin P.T., Maleszewski J.J., Stone J.R. (2020). Pathological features of COVID-19-associated myocardial injury: A multicentre cardiovascular pathology study. Eur. Heart J..

[7] Sheth A.R., Grewal U.S., Patel H.P., Thakkar S., Garikipati S., Gaddam J., Bawa D. (2020). Possible mechanisms responsible for acute coronary events in COVID-19. Med. Hypotheses.

[8] Guan W.J., Ni Z.Y., Hu Y., Liang W.H., Ou C.Q., He J.X., Liu L., Shan H., Lei C.L., Hui D.S.C. (2020). Clinical characteristics of coronavirus disease 2019 in China. N. Engl. J. Med..

[9] Shaw A.C., Goldstein D.R., Montgomery R.R. (2013). Age-dependent dysregulation of innate immunity. Nat. Rev. Immunol..

[10] Channappanavar R., Fehr A.R., Vijay R., Mack M., Zhao J., Meyerholz D.K., Perlman S. (2016). Dysregulated Type I interferon and inflammatory monocyte-macrophage responses cause lethal pneumonia in SARS- CoV-infected mice. Cell Host Microbe.

[11] Law H.K., Cheung C.Y., Ng H.Y., Sia S.F., Chan Y.O., Luk W., Nicholls J.M., Peiris J.S., Lau Y.L. (2005). Chemokine up-regulation in SARS-coronavirus- infected, monocyte-derived human dendritic cells. Blood.

[12] Lau S.K.P., Lau C.C.Y., Chan K.H., Li C.P.Y., Chen H., Jin D.Y., Chan J.F.W., Woo P.C.Y., Yuen K.Y. (2013). Delayed induction of proinflammatory cytokines and suppression of innate antiviral response by the novel Middle East respiratory syndrome coronavirus: Implications for pathogenesis and treatment. J. Gen. Virol..

[13] Jain U. (2020). Effect of COVID-19 on the Organs. Cureus.

[14] Ramcharan T., Nolan O., Lai C.Y., Prabhu N., Krishnamurthy R., Richter A.G., Jyothish D., Kanthimathinathan H.K., Welch S.B., Hackett S. (2020). Paediatric Inflammatory Multisystem Syndrome: Temporally Associated with SARS-CoV-2 (PIMS-TS): Cardiac Features, Management and Short-Term Outcomes at a UK Tertiary Paediatric Hospital. Pediatr. Cardiol..

[15] Gkoutzourelas A., Bogdanos D.P., Sakkas L.I. (2020). Kawasaki Disease and COVID-19. Mediterr. J. Rheumatol..

[16] Wang H., Ma S. (2008). The cytokine storm and factors determining the sequence and severity of organ dysfunction in multiple organ dysfunction syndrome. Am. J. Emerg. Med..

[17] Frydrych L.M., Bian G., O’Lone D.E., Ward P.A., Delano M.J. (2018). Obesity and type 2 diabetes mellitus drive immune dysfunction, infection development, and sepsis mortality. J. Leukoc. Biol..

[18] Giamarellos-Bourboulis E.J., Netea M.G., Rovina N., Akinosoglou K., Antoniadou A., Antonakos N., Damoraki G., Gkavogianni T., Adami M.E., Katsaounou P. (2020). Complex Immune Dysregulation in COVID-19 Patients with Severe Respiratory Failure. Cell Host Microbe.

[19] Siu K.L., Yuen K.S., Castaño-Rodriguez C., Ye Z.W., Yeung M.L., Fung S.Y., Yuan S., Chan C.P., Yuen K.Y., Enjuanes L. (2019). Severe acute respiratory syndrome coronavirus ORF3a protein activates the NLRP3 inflammasome by promoting TRAF3-dependent ubiquitination of ASC. FASEB J..

[20] Costela-Ruiz V.J., Illescas-Montes R., Puerta-Puerta J.M., Ruiz C., Melguizo-Rodríguez L. (2020). SARS-CoV-2 infection: The role of cytokines in COVID-19 disease. Cytokine Growth Factor Rev..

[21] Huang C., Wang Y., Li X., Ren L., Zhao J., Hu Y., Zhang L., Fan G., Xu J., Gu X. (2020). Clinical features of patients infected with 2019 novel coronavirus in Wuhan, China. Lancet.

[22] Zhang W., Zhao Y., Zhang F., Wang Q., Li T., Liu Z., Wang J., Qin Y., Zhang X., Yan X. (2020). The use of anti-inflammatory drugs in the treatment of people with severe coronavirus disease 2019 (COVID-19): The perspectives of clinical immunologists from China. Clin. Immunol..

[23] Blanco-Melo D., Nilsson-Payant B.E., Liu W.C., Uhl S., Hoagland D., Møller R., Jordan T.X., Oishi K., Panis M., Sachs D. (2020). Imbalanced Host Response to SARS-CoV-2 Drives Development of COVID-19. Cell.

[24] Sattar N. (2004). Inflammation and endothelial dysfunction: Intimate companions in the pathogenesis of vascular disease?. Clin. Sci..

[25] Varga Z., Flammer A.J., Steiger P., Haberecker M., Andermatt R., Zinkernagel A.S., Mehra M.R., Schuepbach R.A., Ruschitzka F., Moch H. (2020). Endothelial cell infection and endotheliitis in COVID-19. Lancet.

[26] Korakas E., Ikonomidis I., Kousathana F., Balampanis K., Kountouri A., Raptis A., Palaiodimou L., Kokkinos A., Lambadiari V. (2020). Obesity and COVID-19: Immune and metabolic derangement as a possible link to adverse clinical outcomes. Am. J. Physiol. Endocrinol. Metab..

[27] Lim S., Bae J.H., Kwon H.S., Nauck M.A. (2021). COVID-19 and diabetes mellitus: From pathophysiology to clinical management. Nat. Rev. Endocrinol..

[28] Huang I., Lim M.A., Pranata R. (2020). Diabetes mellitus is associated with increased mortality and severity of disease in COVID-19 pneumonia—A systematic review, meta-analysis, and meta-regression. Diabetes Metab. Syndr..

[29] Kumar A., Arora A., Sharma P., Anikhindi S.A., Bansal N., Singla V., Khare S., Srivastava A. (2020). Is diabetes mellitus associated with mortality and severity of COVID-19? A meta-analysis. Diabetes Metab. Syndr..

[30] Shang L., Shao M., Guo Q., Shi J., Zhao Y., Xiaokereti J., Tang B. (2020). Diabetes Mellitus is Associated with Severe Infection and Mortality in Patients with COVID-19: A Systematic Review and Meta-analysis. Arch. Med. Res..

[31] Bello-Chavolla O.Y., Bahena-López J.P., Antonio-Villa N.E., Vargas-Vázquez A., González-Díaz A., Márquez-Salinas A., Fermín-Martínez C.A., Naveja J.J., Aguilar-Salinas C.A. (2020). Predicting Mortality Due to SARS-CoV-2: A Mechanistic Score Relating Obesity and Diabetes to COVID-19 Outcomes in Mexico. J. Clin. Endocrinol. Metab..

[32] Huang Y., Lu Y., Huang Y.M., Wang M., Ling W., Sui Y., Zhao H.L. (2020). Obesity in patients with COVID-19: A systematic review and meta-analysis. Metabolism.

[33] Yang J., Ma Z., Lei Y. (2020). A meta-analysis of the association between obesity and COVID-19. Epidemiol. Infect..

[34] Zhao X., Gang X., He G., Li Z., Lv Y., Han Q., Wang G. (2020). Obesity Increases the Severity and Mortality of Influenza and COVID-19: A Systematic Review and Meta-Analysis. Front. Endocrinol..

[35] Li X., Guan B., Su T., Liu W., Chen M., Bin Waleed K., Guan X., Gary T., Zhu Z. (2020). Impact of cardiovascular disease and cardiac injury on in-hospital mortality in patients with COVID-19: A systematic review and meta-analysis. Heart.

[36] Ssentongo P., Ssentongo A.E., Heilbrunn E.S., Ba D.M., Chinchilli V.M. (2020). Association of cardiovascular disease and 10 other pre-existing comorbidities with COVID-19 mortality: A systematic review and meta-analysis. PLoS ONE.

[37] Inciardi R.M., Lupi L., Zaccone G., Italia L., Raffo M., Tomasoni D., Cani D.S., Cerini M., Farina D., Gavazzi E. (2020). Cardiac Involvement in a Patient With Coronavirus Disease 2019 (COVID-19). JAMA Cardiol..

[38] Babapoor-Farrokhran S., Gill D., Walker J., Rasekhi R.T., Bozorgnia B., Amanullah A. (2020). Myocardial injury and COVID-19: Possible mechanisms. Life Sci..

[39] Ranard L.S., Fried J.A., Abdalla M., Anstey D.E., Givens R.C., Kumaraiah D., Kodali S.K., Takeda K., Karmpaliotis D., Rabbani L.E. (2020). Approach to Acute Cardiovascular Complications in COVID-19 Infection. Circ. Heart Fail..

[40] Kwenandar F., Japar K.V., Damay V., Hariyanto T.I., Tanaka M., Lugito N.P.H., Kurniawan A. (2020). Coronavirus disease 2019 and cardiovascular system: A narrative review. Int. J. Cardiol. Heart Vasc..

[41] Inciardi R.M., Adamo M., Lupi L., Cani D.S., Di Pasquale M., Tomasoni D., Italia L., Zaccone G., Tedino C., Fabbricatore D. (2020). Characteristics and outcomes of patients hospitalized for COVID-19 and cardiac disease in Northern Italy. Eur. Heart J..

[42] Wolfler A., Manarino S., Giacomet V., Camporesi A., Zuccotti G. (2020). Acute myocardial injury: A novel clinical pattern in children with COVID-19. Lancet Child. Adolesc. Health.

[43] Toubiana J., Poirault C., Corsia A., Bajolle F., Fourgeaud J., Angoulvant F., Debray A., Basmaci R., Salvador E., Biscardi S. (2020). Kawasaki-like multisystem inflammatory syndrome in children during the covid-19 pandemic in Paris, France: Prospective observational study. BMJ.

[44] Valverde I., Singh Y., Sanchez-de-Toledo J., Theocharis P., Chikermane A., Di Filippo S., Kuciñska B., Mannarino S., Tamariz-Martel A., Gutierrez-Larraya F. (2021). Acute Cardiovascular Manifestations in 286 Children With Multisystem Inflammatory Syndrome Associated With COVID-19 Infection in Europe. Circulation.

[45] Amirfakhryan H. (2020). Kawasaki-like disease in children with COVID-19: A hypothesis. Med. Hypotheses.

[46] Eberli F.R., Kurz D. (2020). Cardiovascular aspects of COVID-19. Swiss Med. Wkly..

[47] Whittaker E., Bamford A., Kenny J., Kaforou M., Jones C.E., Shah P., Ramnarayan P., Fraisse A., Miller O., Davies P. (2020). Clinical Characteristics of 58 Children With a Pediatric Inflammatory Multisystem Syndrome Temporally Associated With SARS-CoV-2. JAMA.

[48] Feldstein L.R., Rose E.B., Horwitz S.M., Collins J.P., Newhams M.M., Son M.B.F., Newburger J.W., Kleinman L.C., Heidemann S.M., Martin A.A. (2020). Multisystem Inflammatory Syndrome in U.S. Children and Adolescents. N. Engl. J. Med..

[49] Carlin R.F., Fischer A.M., Pitkowsky Z., Abel D., Sewell T.B., Landau E.G., Caddle S., Robbins-Milne L., Boneparth A., Milner J.D. (2021). Discriminating Multisystem Inflammatory Syndrome in Children Requiring Treatment from Common Febrile Conditions in Outpatient Settings. J. Pediatr..

[50] Sharma M., Gorstein S., Aldrich M.L., Hsu D.T., Choueiter N.F. (2020). Reversible Myocardial Injury Associated With SARS-CoV-2 in an Infant. JACC Case Rep..

[51] Davies P., Evans C., Kanthimathinathan H.K., Lillie J., Brierley J., Waters G., Johnson M., Griffiths B., du Pré P., Mohammad Z. (2020). Intensive care admissions of children with paediatric inflammatory multisystem syndrome temporally associated with SARS-CoV-2 (PIMS-TS) in the UK: A multicentre observational study. Lancet Child. Adolesc. Health.

[52] Oleszak F., Maryniak A., Botti E., Abrahim C., Salifu M.O., Youssef M., Henglein V.L., McFarlane S.I. (2020). Myocarditis Associated With COVID-19. Am. J. Med. Case Rep..

[53] Licu R.A., Blîndu E., Opincariu D., Benedek T. (2020). Vulnerable Plaques Producing an Acute Coronary Syndrome Exhibit a Different CT Phenotype than Those That Remain Silent. J. Cardiovasc. Emergencies.

[54] Lazou A., Ikonomidis I., Bartekova M., Benedek T., Makavos G., Palioura D., Cabrera Fuentes H., Andreadou I. (2020). Chronic inflammatory diseases, myocardial function and cardioprotection. Br. J. Pharmacol..

[55] Benedek T. (2020). COVID-19 Pandemic and Cardiovascular Challenges. J. Cardiovasc. Emergencies.

[56] Benedek T., Rodean I., Ratiu M., Rat N., Yero Eremie L., Biris C., Lazar L., Pacurar M., Benedek I. (2018). Periodontal Disease, Infammation and Atherosclerosis Progression in Patients with Acute Coronary Syndromes—the ATHERODENT Study. J. Cardiovasc. Emergencies.

[57] Rus V.A., Chitu M., Cernea S., Benedek I., Hodas R., Zavate R., Nyulas T., Hintea M., Benedek T. (2020). Altered nutritional status, inflammation and systemic vulnerability in patients with acute myocardial infarction undergoing percutaneous coronary revascularisation: A prospective study in a level 3 cardiac critical care unit. Nutr. Diet..

[58] Malavazos A.E., Goldberger J.J., Iacxobellis G. (2020). Does epicardial fat contribute to COVID-19 myocardial inflammation?. Eur. Heart J..

[59] Rus A., Rat N., Chitu M., Benedek I. (2020). The Characteristics, Manifestations and Cardiopulmonary Imaging (CT/MRI) of COVID-19 in COVID-19 Patients. J. Interdiscip. Med..

[60] Libenciuc C., Licu R.A., Hodas R., Chitu M., Benedek I. (2020). Myocardial Injury and Myocarditis in SARS-CoV-2 Patients. J. Interdiscip. Med..

[61] Biasucci L.M., Leo M., De Maria G.L. (2008). Local and systemic mechanisms of plaque rupture. Angiology.

[62] Mauriello A., Sangiorgi G., Fratoni S., Palmieri G., Bonanno E., Anemona L., Schwartz R.S., Spagnoli L.G. (2005). Diffuse and active inflammation occurs in both vulnerable and stable plaques of the entire coronary tree: A histopathologic study of patients dying of acute myocardial infarction. J. Am. Coll. Cardiol..

[63] Schiavone M., Gasperetti A., Mancone M. (2020). Redefining the prognostic value of high-sensitivity troponin in COVID-19 patients: The importance of concomitant coronary artery disease. J. Clin. Med..

[64] Galván-Román J.M., Rodríguez-García S.C., Roy-Vallejo E., Marcos-Jiménez A., Sánchez-Alonso S., Fernández-Díaz C., Alcaraz-Serna A., Mateu-Albero T., Rodríguez-Cortes P., Sánchez-Cerrillo I. (2021). IL-6 serum levels predict severity and response to tocilizumab in COVID-19: An observational study. J. Allergy Clin. Immunol..

[65] Liu F., Li L., Xu M., Wu J., Luo D., Zhu Y., Li B., Song X., Zhou X. (2020). Prognostic value of interleukin-6, C-reactive protein, and procalcitonin in patients with COVID-19. J. Clin. Virol..

[66] Gao Y., Li T., Han M., Li X., Wu D., Xu Y., Zhu Y., Liu Y., Wang X., Wang L. (2020). Diagnostic utility of clinical laboratory data determinations for patients with the severe COVID-19. J. Med. Virol..

[67] Qin C., Zhou L., Hu Z., Zhang S., Yang S., Tao Y., Xie C., Ma K., Shang K., Wang W. (2020). Dysregulation of Immune Response in Patients with Coronavirus 2019 (COVID-19) in Wuhan, China. Clin. Infect. Dis..

[68] Henry B.M., de Oliveira M.H.S., Benoit S., Plebani M., Lippi G. (2020). Hematologic, biochemical and immune biomarker abnormalities associated with severe illness and mortality in coronavirus disease 2019 (COVID-19): A meta-analysis. Clin. Chem. Lab. Med..

[69] Coomes E.A., Haghbayan H. (2020). Interleukin-6 in Covid-19: A systematic review and meta-analysis. Rev. Med Virol..

[70] Zeng F., Huang Y., Guo Y., Yin M., Chen X., Xiao L., Deng G. (2020). Association of inflammatory markers with the severity of COVID-19: A meta-analysis. Int. J. Infect. Dis..

[71] Akbari H., Tabrizi R., Lankarani K.B., Aria H., Vakili S., Asadian F., Noroozi S., Keshavarz P., Faramarz S. (2020). The role of cytokine profile and lymphocyte subsets in the severity of coronavirus disease 2019 (COVID-19): A systematic review and meta-analysis. Life Sci..

[72] Mo P., Xing Y., Xiao Y., Deng L., Zhao Q., Wang H., Xiong Y., Cheng Z., Gao S., Liang K. (2020). Clinical characteristics of refractory COVID-19 pneumonia in Wuhan, China. Clin. Infect. Dis..

[73] Zhang J.J., Dong X., Cao Y.Y., Yuan Y.D., Yang Y.B., Yan Y.Q., Akdis C.A., Gao Y.D. (2020). Clinical characteristics of 140 patients infected with SARS-CoV-2 in Wuhan, China. Allergy.

[74] Cordeanu E.M., Duthil N., Severac F., Lambach H., Tousch J., Jambert L., Mirea C., Delatte A., Younes W., Frantz A.S. (2020). Prognostic Value of Troponin Elevation in COVID-19 hospitalized patients. J. Clin. Med..

[75] Barman H.A., Atici A., Sahin I., Alici G., Tekin E.A., Baycan Ö.F., Ozturk F., Oflar E., Tugrul S., Yavuz M.B. (2020). Prognostic significance of cardiac injury in COVID-19 patients wuth and without coronary artery disease. Coron. Artery Dis..

[76] Peterson E., Lo K.B., DeJoy R., Salacup G., Pelayo J., Bhargav R., Gul F., Albano J., Azmaiparashvili Z., Amanullah A. (2020). The relationship between coronary artery disease and clinical outcomes in COVID-19: A single-center retrospective analysis. Coron. Artery Dis..

[77] Grzegorowska O., Lorkowski J. (2020). Possible correlations between atherosclerosis, acute coronary syndromes and COVID-19. J. Clin. Med..

[78] Zhou F., Yu T., Du R., Fan G., Liu Y., Liu Z., Xiang J., Wang Y., Song B., Gu X. (2020). Clinical course and risk factors for mortality of adult inpatients with COVID-19 in Wuhan, China: A retrospective cohort study. Lancet.

[79] Chen C., Huihui L., Hang W., Wang D.W. (2020). Cardiac injuries in coronavirus disease 2019 (COVID-19). J. Mol. Cell Cardiol..

[80] Han H., Xie L., Liu R., Yang J., Liu F., Wu K., Chen L., Hou W., Feng Y., Zhu C. (2020). Analysis of heart injury laboratory parameters in 273 COVID-19 patients in one hospital in Wuhan, China. J. Med. Virol..

[81] Bikdeli B., Madhavan M.V., Jimenez D., Chuich T., Dreyfus I., Driggin E., Nigoghossian C., Ageno W., Madjid M., Guo Y. (2020). COVID-19 and Thrombotic of Thromboembolic Disease: Implications for prevention, antithrombotic Therapy and Follow-Up. J. Am. Coll. Cardiol..

[82] Grobler C., Maphumulo S.C., Grobbelaar L.M. (2020). Covid-19: The Rollercoaster of fibrin(Ogen), D-Dimer, Von Willebrand Factor, P-Selectin and their interactions with endothelial cells, platelets and erythrocytes. Int. J. Mol. Sci..

[83] Rodriguez-Diez R.R., Tejera-Muñoz A., Marquez-Exposito L., Rayego-Mateos S., Santos Sanchez L., Marchant V., Tejedor Santamaria L., Ramos A.M., Ortiz A., Egido J. (2020). Statins: Could and old friend help in the fight against COVID-19?. Br. J. Pharmacol..

[84] Subir R., Jagat M., Kalyan G. (2020). Pros and cons for use of statins in people with coronavirus disease-19. Diabetes Metab. Syndr..

[85] De Spiegeleer A., Bronselaer A., Teo J.T., Byttebier G., De Tré G., Belmans L., Dobson R., Wynendaele E., Van De Wiele C., Vandaele F. (2020). The effects of ARBs, ACEis, and Statins on Clinical Outcomes of COVID-19 Infection Among Nursing Home Residents. JAMDA.

[86] Mittachione G., Schiavone M., Curnis A., Arca M., Antinori S., Gasperetti A., Mascioli G., Severino P., Sabato F., Caracciolo M.M. (2021). Impact of prior statin use on clinical outcomes in COVID-19 patients: Data from tertiary referral hospitals during COVID-19 pandemic in Italy. J. Clin. Lipidol..

[87] Cernea S., Dobreanu M. (2013). Diabetes and beta cell function: From mechanisms to evaluation and clinical implications. Biochem. Med..

[88] Prattichizzo F., De Nigris V., Spiga R., Mancuso E., La Sala L., Antonicelli R., Testa R., Procopio A.D., Olivieri F., Ceriello A. (2018). Inflammageing and metaflammation: The yin and yang of type 2 diabetes. Ageing Res. Rev..

[89] Hotamisligil G.S. (2017). Inflammation, metaflammation and immunometabolic disorders. Nature.

[90] Hotamisligil G.S., Peraldi P., Budavari A., Ellis R., White M.F., Spiegelman B.M. (1996). IRS-1-mediated inhibition of insulin receptor tyrosine kinase activity in TNF-alpha- and obesity-induced insulin resistance. Science.

[91] Bastard J.P., Maachi M., Van Nhieu J.T., Jardel C., Bruckert E., Grimaldi A., Robert J.J., Capeau J., Hainque B. (2002). Adipose tissue IL-6 content correlates with resistance to insulin activation of glucose uptake both in vivo and in vitro. J. Clin. Endocrinol. Metab..

[92] Bastard J.P., Maachi M., Lagathu C., Kim M.J., Caron M., Vidal H., Capeau J., Feve B. (2006). Recent advances in the relationship between obesity, inflammation, and insulin resistance. Eur. Cytokine Netw..

[93] Kanda H., Tateya S., Tamori Y., Kotani K., Hiasa K., Kitazawa R., Kitazawa S., Miyachi H., Maeda S., Egashira K. (2006). MCP-1 contributes to macrophage infiltration into adipose tissue, insulin resistance, and hepatic steatosis in obesity. J. Clin. Investig..

[94] Chylikova J., Dvorackova J., Tauber Z., Kamarad V. (2018). M1/M2 macrophage polarization in human obese adipose tissue. Biomed. Pap. Med. Fac. Univ. Palacky Olomouc. Czech. Repub..

[95] Zhu L., Zhao Q., Yang T., Ding W., Zhao Y. (2015). Cellular metabolism and macrophage functional polarization. Int. Rev. Immunol..

[96] Tannahill G.M., Curtis A.M., Adamik J., Palsson-McDermott E.M., McGettrick A.F., Goel G., Frezza C., Bernard N.J., Kelly B., Foley N.H. (2013). Succinate is an inflammatory signal that induces IL-1β through HIF-1α. Nature.

[97] Galván-Peña S., O’Neill L.A. (2014). Metabolic reprograming in macrophage polarization. Front. Immunol..

[98] Soto-Heredero G., Gómez de Las Heras M.M., Gabandé-Rodríguez E., Oller J., Mittelbrunn M. (2020). Glycolysis—A key player in the inflammatory response. FEBS J..

[99] Michalek R.D., Gerriets V.A., Jacobs S.R., Macintyre A.N., MacIver N.J., Mason E.F., Sullivan S.A., Nichols A.G., Rathmell J.C. (2011). Cutting edge: Distinct glycolytic and lipid oxidative metabolic programs are essential for effector and regulatory CD4+ T cell subsets. J. Immunol..

[100] Johnson A.R., Milner J.J., Makowski L. (2012). The inflammation highway: Metabolism accelerates inflammatory traffic in obesity. Immunol. Rev..

[101] Thiem K., Keating S.T., Netea M.G., Riksen N.P., Tack C.J., van Diepen J., Stienstra R. (2021). Hyperglycemic Memory of Innate Immune Cells Promotes In Vitro Proinflammatory Responses of Human Monocytes and Murine Macrophages. J. Immunol..

[102] Lachmandas E., Vrieling F., Wilson L.G., Joosten S.A., Netea M.G., Ottenhoff T.H., van Crevel R. (2015). The effect of hyperglycaemia on in vitro cytokine production and macrophage infection with Mycobacterium tuberculosis. PLoS ONE.

[103] Dror E., Dalmas E., Meier D.T., Wueest S., Thévenet J., Thienel C., Timper K., Nordmann T.M., Traub S., Schulze F. (2017). Postprandial macrophage-derived IL-1β stimulates insulin, and both synergistically promote glucose disposal and inflammation. Nat. Immunol..

[104] van Diepen J.A., Thiem K., Stienstra R., Riksen N.P., Tack C.J., Netea M.G. (2016). Diabetes propels the risk for cardiovascular disease: Sweet monocytes becoming aggressive?. Cell Mol. Life Sci..

[105] Abbadi A., Loftis J., Wang A., Yu M., Wang Y., Shakya S., Li X., Maytin E., Hascall V. (2020). Heparin inhibits proinflammatory and promotes anti-inflammatory macrophage polarization under hyperglycemic stress. J. Biol. Chem..

[106] Nishikawa T., Edelstein D., Du X.L., Yamagishi S., Matsumura T., Kaneda Y., Yorek M.A., Beebe D., Oates P.J., Hammes H.P. (2000). Normalizing mitochondrial superoxide production blocks three pathways of hyperglycaemic damage. Nature.

[107] Guzik T.J., Cosentino F. (2018). Epigenetics and Immunometabolism in Diabetes and Aging. Antioxid. Redox Signal..

[108] Chen J., Brodsky S.V., Goligorsky D.M., Hampel D.J., Li H., Gross S.S., Goligorsky M.S. (2002). Glycated collagen I induces premature senescence-like phenotypic changes in endothelial cells. Circ. Res..

[109] Rogero M.M., Calder P.C. (2018). Obesity, Inflammation, Toll-Like Receptor 4 and Fatty Acids. Nutrients.

[110] Lee J.Y., Sohn K.H., Rhee S.H., Hwang D. (2001). Saturated fatty acids, but not unsaturated fatty acids, induce the expression of cyclooxygenase-2 mediated through Toll-like receptor 4. J. Biol. Chem..

[111] Hwang D.H., Kim J.A., Lee J.Y. (2016). Mechanisms for the activation of Toll-like receptor 2/4 by saturated fatty acids and inhibition by docosahexaenoic acid. Eur. J. Pharmacol..

[112] Reynolds C.M., McGillicuddy F.C., Harford K.A., Finucane O.M., Mills K.H., Roche H.M. (2012). Dietary saturated fatty acids prime the NLRP3 inflammasome via TLR4 in dendritic cells-implications for diet-induced insulin resistance. Mol. Nutr. Food Res..

[113] Huang S., Rutkowsky J.M., Snodgrass R.G., Ono-Moore K.D., Schneider D.A., Newman J.W., Adams S.H., Hwang D.H. (2012). Saturated fatty acids activate TLR-mediated proinflammatory signaling pathways. J. Lipid Res..

[114] Charles-Messance H., Mitchelson K.A.J., De Marco Castro E., Sheedy F.J., Roche H.M. (2020). Regulating metabolic inflammation by nutritional modulation. J. Allergy Clin. Immunol..

[115] Tilg H., Zmora N., Adolph T.E., Elinav E. (2020). The intestinal microbiota fuelling metabolic inflammation. Nat. Rev. Immunol..

[116] Duewell P., Kono H., Rayner K.J., Sirois C.M., Vladimer G., Bauernfeind F.G., Abela G.S., Franchi L., Nuñez G., Schnurr M. (2010). NLRP3 inflammasomes are required for atherogenesis and activated by cholesterol crystals. Nature.

[117] Jiang Y., Wang M., Huang K., Zhang Z., Shao N., Zhang Y., Wang W., Wang S. (2012). Oxidized low-density lipoprotein induces secretion of interleukin-1β by macrophages via reactive oxygen species-dependent NLRP3 inflammasome activation. Biochem. Biophys. Res. Commun..

[118] Bekkering S., Quintin J., Joosten L.A., van der Meer J.W., Netea M.G., Riksen N.P. (2014). Oxidized low-density lipoprotein induces long-term proinflammatory cytokine production and foam cell formation via epigenetic reprogramming of monocytes. Arterioscler. Thromb. Vasc. Biol..

[119] Rajani C., Jia W. (2018). Disruptions in gut microbial-host co-metabolism and the development of metabolic disorders. Clin. Sci..

[120] Schroder K., Sagulenko V., Zamoshnikova A., Richards A.A., Cridland J.A., Irvine K.M., Stacey K.J., Sweet M.J. (2012). Acute lipopolysaccharide priming boosts inflammasome activation independently of inflammasome sensor induction. Immunobiology.

[121] Amar J., Chabo C., Waget A., Klopp P., Vachoux C., Bermúdez-Humarán L.G., Smirnova N., Bergé M., Sulpice T., Lahtinen S. (2011). Intestinal mucosal adherence and translocation of commensal bacteria at the early onset of type 2 diabetes: Molecular mechanisms and probiotic treatment. EMBO Mol. Med..

[122] Thaiss C.A., Levy M., Grosheva I., Zheng D., Soffer E., Blacher E., Braverman S., Tengeler A.C., Barak O., Elazar M. (2018). Hyperglycemia drives intestinal barrier dysfunction and risk for enteric infection. Science.

[123] Sell H., Habich C., Eckel J. (2012). Adaptive immunity in obesity and insulin resistance. Nat. Rev. Endocrinol..

[124] Feldman E.L., Savelieff M.G., Hayek S.S., Pennathur S., Kretzler M., Pop-Busui R. (2020). COVID-19 and Diabetes: A Collision and Collusion of Two Diseases. Diabetes.

[125] Brufsky A. (2020). Hyperglycemia, hydroxychloroquine, and the COVID-19 pandemic. J. Med. Virol..

[126] Bode B., Garrett V., Messler J., McFarland R., Crowe J., Booth R., Klonoff D.C. (2020). Glycemic Characteristics and Clinical Outcomes of COVID-19 Patients Hospitalized in the United States. J. Diabetes Sci. Technol..

[127] Suleyman G., Fadel R.A., Malette K.M., Hammond C., Abdulla H., Entz A., Demertzis Z., Hanna Z., Failla A., Dagher C. (2020). Clinical Characteristics and Morbidity Associated with Coronavirus Disease 2019 in a Series of Patients in Metropolitan Detroit. JAMA Netw. Open.

[128] Petrilli C.M., Jones S.A., Yang J., Rajagopalan H., O’Donnell L., Chernyak Y., Tobin K.A., Cerfolio R.J., Francois F., Horwitz L.I. (2020). Factors associated with hospital admission and critical illness among 5279 people with coronavirus disease 2019 in New York City: Prospective cohort study. BMJ.

[129] Zhu L., She Z.G., Cheng X., Qin J.J., Zhang X.J., Cai J., Lei F., Wang H., Xie J., Wang W. (2020). Association of Blood Glucose Control and Outcomes in Patients with COVID-19 and Pre-existing Type 2 Diabetes. Cell Metab..

[130] Chao W.C., Tseng C.H., Wu C.L., Shih S.J., Yi C.Y., Chan M.C. (2020). Higher glycemic variability within the first day of ICU admission is associated with increased 30-day mortality in ICU patients with sepsis. Ann. Intensive Care.

